# Numerical Evaluation of a Zero Poisson’s Ratio Structure in µ-3D-Printed Self-Expanding Nitinol Stents

**DOI:** 10.3390/mi17060736

**Published:** 2026-06-18

**Authors:** Farhana Yasmin, Ana Vafadar, Majid Tolouei-Rad

**Affiliations:** 1 Center for Advanced Materials and Manufacturing, School of Engineering, Edith Cowan University (ECU), 270 Joondalup Drive, Joondalup, WA 6027, Australia; 2 Mineral Recovery Research Centre, School of Engineering, Edith Cowan University (ECU), 270 Joondalup Drive, Joondalup, WA 6027, Australia; a.vafadar@ecu.edu.au

**Keywords:** self-expanding stent, Nitinol, positive Poisson’s ratio (PPR), zero Poisson’s ratio (ZPR), negative Poisson’s ratio (NPR), 3D printing

## Abstract

Stenting is a minimally invasive treatment used in managing peripheral artery disease (PAD). However, clinical challenges persist, including in-stent thrombosis and restenosis, primarily driven by axial foreshortening or elongation and suboptimal balance between radial stiffness and flexibility inherent to conventional stent designs. This study proposes an innovative arrow-shaped geometry exhibiting zero Poisson’s ratio (ZPR) behaviour for 3D-printed self-expanding Nitinol stents. The complete stent deployment process was modelled using finite element analysis (FEA), including radial crimping and subsequent expansion to enable systematic parametric investigation while accounting for µ-3D printing constraints. Response surface methodology (RSM) rigorously evaluated mechanical performance, defining peak stress, chronic outward force (COF), radial resistive force (RRF), and foreshortening (FS) as constraint and objective functions within the optimisation framework. The optimised ZPR stent achieved favourable performance: extremely low foreshortening (|FS| ≤ 0.12%), representing outstanding axial stability compared with previously reported self-expanding stents, and a well-balanced radial response with ~50% higher radial strength than positive Poisson’s ratio (PPR) structures, while 16.67% lower than negative Poisson’s ratio (NPR) counterparts. These results highlight the ZPR stent’s capability to minimise axial deformation while maintaining adequate radial support, highlighting substantial potential for precise, stable deployment in PAD applications.

## 1. Introduction

### 1.1. Background

**Figure 1:**
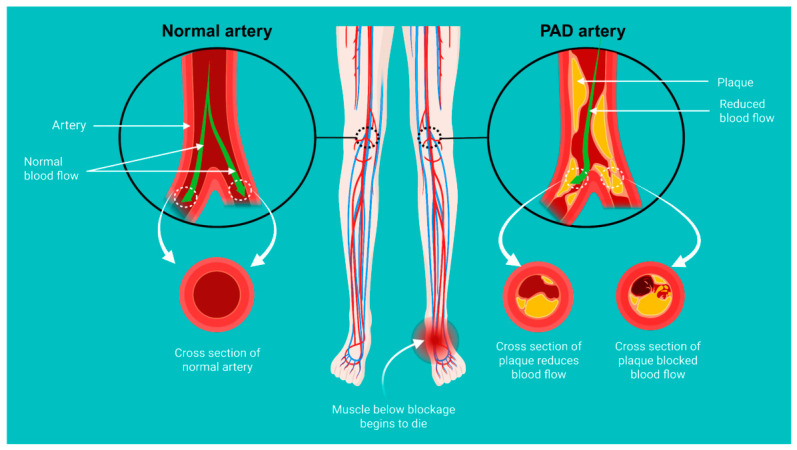
Schematic diagram of peripheral artery disease (PAD).

Peripheral artery disease (PAD) predominantly affects older adults, particularly those aged 65 and above. As shown for 2024, PAD impacts an estimated 200 million people globally, with 20% to 30% of diagnosed patients expected to die within five years [1,2]. The most frequently affected regions are the arteries of the lower limb, especially the popliteal artery (PA) and superficial femoral artery (SFA). PAD typically results from atherosclerosis, a condition characterised by the buildup of plaque on the inner arterial walls (Figure 1). This buildup leads to arterial narrowing or blockage, restricting blood circulation and reducing oxygen delivery necessary for normal metabolic function in the lower limbs. Consequently, patients may experience symptoms such as discomfort, pain, and intermittent claudication during physical activity, along with an increased risk of more severe complications like heart disease and stroke [3].

Stent implantation has become an increasingly preferred treatment option in recent years due to its documented benefits, such as faster recovery, reduced pain, and minimal scarring [4,5]. An ideal stent should provide sufficient radial strength to support the vessel wall, ensure reliable scaffolding after expansion, and achieve substantial radial expansion (typically 90–100%) to restore blood flow. Additionally, a stent should exhibit minimal radial and axial recoil, negligible foreshortening, excellent biocompatibility, consistent deformation during deployment, and high fatigue resistance [6,7,8]. Stents are typically classified into two types: balloon-expanding and self-expanding stents. Balloon-expanding stents function by inducing plastic deformation through balloon inflation, whereas self-expanding stents rely on the superelastic behaviour or transition temperature of materials, such as shape memory alloys (SMA), to achieve expansion. Compared to traditional balloon angioplasty, self-expanding stents offer the distinct advantage of recovering their original shape even after complete radial collapse or compression [9,10].

To date, Nitinol (NiTi-based alloy) is the most widely used smart material in Food and Drug Administration (FDA)-approved self-expanding stent systems, such as *s.m.a.r.t* and *Intracoil* stents [11,12]. Further, its unique combination of exceptional superelastic behaviour, shape memory effect, superior fatigue durability, and excellent biocompatibility makes it an ideal candidate for SFA arteries [13]. However, self-expanding Nitinol stents generally exhibit lower radial force, making them more susceptible to arterial recoil and in-stent restenosis [14,15]. To compensate, stents are often oversized, which can induce excessive mechanical stress on the vessel wall and lead to adverse biological responses such as thrombosis and neointimal hyperplasia [16,17]. Unlike balloon-expandable stents, self-expanding stents are fabricated in their fully expanded form and undergo large reversible deformation during crimping and deployment, thereby imposing more stringent structural requirements on the stent design.

### 1.2. Current Designs and Limitations

Assessing and comparing various stent geometries allows clinicians to identify the most appropriate and effective design for individual patient needs [18]. Besides this, finite element analysis (FEA) of Nitinol-based stents minimises the need for physical prototyping and accelerates product development by enabling virtual simulation of arterial deployment [19,20,21,22]. Conventional stent designs typically present a positive Poisson’s ratio (PPR), providing sufficient radial strength and chronic outward force (COF) to support vessel patency [23]. However, these designs suffer from limited flexibility and significant foreshortening during expansion, which compromises deployment accuracy and positioning in complex vascular geometries [24,25,26]. Axial elongation during crimping can lead to mismatched length, causing malapposition in curved arteries [27,28]. For example, hybrid PPR stents investigated by Kim et al. [29] demonstrated improved radial strength compared with the ABSORB stent model (Abbott Vascular, USA) but still exhibited foreshortening exceeding 5%. Conversely, negative Poisson’s ratio (NPR) or auxetic stents expand longitudinally during radial expansion, thereby reducing the risk of migration and improving placement stability [30,31,32,33,34,35]. However, this behaviour introduces axial elongation, which may lead to undesirable vessel interaction. Moreover, many auxetic geometries are unsuitable for self-expanding Nitinol stents due to their structural complexity and instability under large deformation during crimping and expansion [5,30,32,36,37,38,39]. Furthermore, most studies on auxetic stents have focused on polymeric materials or conventional metals, with limited investigation into Nitinol, largely due to fabrication constraints associated with traditional manufacturing techniques, such as laser cutting [40]. In this context, the rise in additive manufacturing (AM), or 3D printing, has introduced a novel and promising route for Nitinol fabrication in recent years, offering increased structural design freedom and the realisation of patient-specific self-expanding stents [41,42,43,44,45].

**Figure 2:**
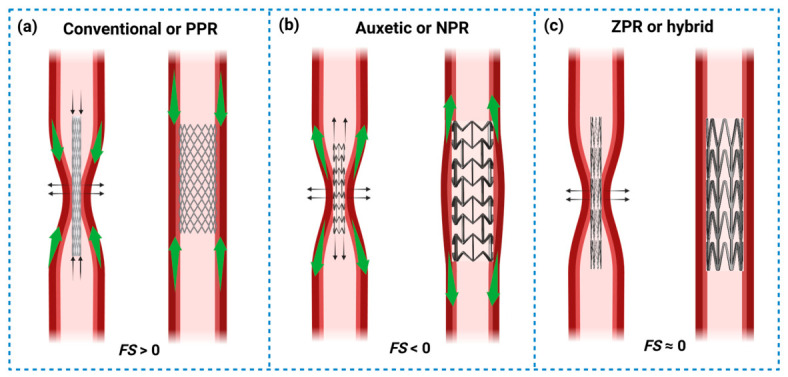
Radial expansion comparison between (**a**) PPR, (**b**) NPR, and (**c**) ZPR structural designs.

In summary, during radial expansion, PPR stents apply inward tension by pulling the vessel wall due to longitudinal foreshortening (FS), narrowing adjacent vessel segments (Figure 2a). In contrast, NPR stents generate outward tension by pushing the vessel wall due to longitudinal elongation, leading to the expansion of nearby vessel areas (Figure 2b) [46]. Both inward and outward frictions can damage the delicate endothelial lining [47,48]. To address these limitations of PPR and NPR structures, zero Poisson’s ratio (ZPR) or hybrid structures (i.e., combining features of both PPR and NPR) have been proposed as a potential solution. These designs enable near-zero axial deformation during radial expansion (Figure 2c), thereby offering the possibility of balancing radial strength, flexibility, and COF while minimising vessel trauma and maintaining uniform lumen patency. Although ZPR and hybrid architectures have been extensively studied in mechanical metamaterials [49,50,51], their application in vascular stents remains limited and insufficiently characterised under large-deformation deployment conditions. In this context, Prithipaul et al. [52] used FEA to compare various unit cell geometries, showing that chevron-type hybrid configurations achieved near-zero foreshortening while maintaining improved radial stiffness and reduced recoil. However, their analysis was limited to periodic unit-cell behaviour and therefore cannot accurately represent patient-specific stent geometries or the non-periodic deformation occurring during deployment. Similarly, Wang et al. [53] proposed a semi-re-entrant honeycomb-based ZPR design for a 3D-printed polymeric vascular stent to investigate its radial strength and bending flexibility. However, the limited flexibility and mechanical performance of the ZPR stent indicate the need for further design optimisation. In addition, their study was conducted in the context of balloon-expandable stents, which do not involve the large deformation associated with crimping and self-expansion.

### 1.3. Study Objectives

This study aims to develop a novel, mechanically viable ZPR architecture for self-expanding Nitinol stents, derived from an NPR metamaterial and tailored for deployment under large deformation. Unlike prior studies that primarily focus on lattice-level analysis, polymer-based systems, or balloon-expandable stents, this work systematically evaluates ZPR-based designs for self-expanding Nitinol stents under realistic deployment conditions. Despite existing research, the mechanical performance of these architectures during deployment, particularly the coupled radial-axial response, remains insufficiently characterised. In this work, a combined computational framework incorporating FEA and RSM is employed to evaluate and optimise the proposed design based on clinically relevant performance metrics, including RRF, COF, FS, and peak stress. The deployment process is explicitly modelled through radial crimping and expansion to capture the non-uniform deformation behaviour associated with superelastic Nitinol. Lastly, the optimised ZPR design is benchmarked against conventional PPR and NPR stent configurations, demonstrating its ability to decouple radial and axial responses and thereby resolve the trade-off between foreshortening and radial mechanical performance.

## 2. Materials and Methods

### 2.1. Materials

**Figure 3:**
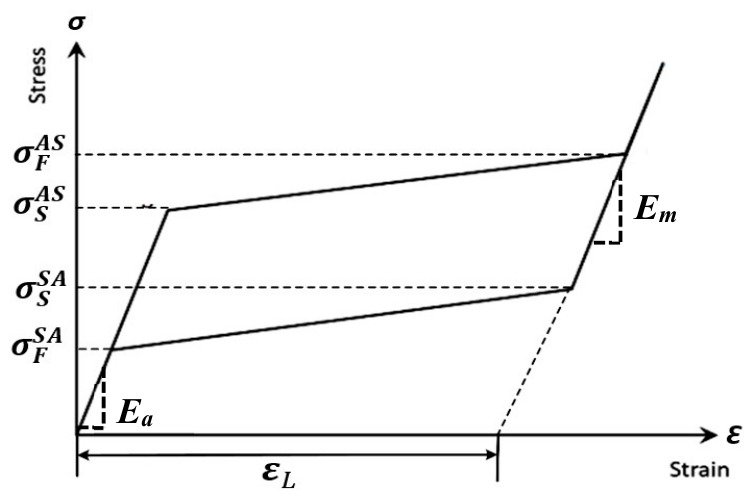
Superelastic model of shape-memory alloy (adopted from Alaimo et al. [55]).

Self-expanding stents were modelled using a superelastic Nitinol material, implemented via the shape memory alloy model provided in ANSYS Workbench 2024 R1 (ANSYS, Inc., Canonsburg, PA, USA). A defining feature of Nitinol is its superelastic behaviour, which permits the material to endure substantial deformations and still return to its initial form once the load is removed, making it exceptionally flexible. Under normal conditions, Nitinol exists in the austenite phase. However, when mechanical stress exceeds a specific threshold $\sigma_{F}^{AS}$ and the temperature surpasses the austenite finish temperature (A_f_), the alloy undergoes a phase transformation from austenite to martensite. This transformation results in significant strain, which is fully recovered upon unloading as the martensite reverts to austenite. Since these transformation strains (approximately 6–7%) significantly exceed typical elastic strains in metals, this behaviour is known as superelasticity [54]. Accordingly, $\sigma_{S}^{AS}$ and $\sigma_{F}^{AS}$ refer to the start and finish stresses, respectively, of the austenite-to-martensite (AS) transformation, while $\sigma_{S}^{SA}$ and $\sigma_{F}^{SA}$ correspond to the start and finish stresses, respectively, of the reverse martensite-to-austenite (SA) transformation, as shown in Figure 3. The parameter *α* accounts for the difference in material response under compression and tension, as defined by Equation (1) [55]:

(1) $$\alpha =\frac{{\left(\sigma_{S}^{AS}\right)}_{c}-{\left(\sigma_{S}^{AS}\right)}_{t}}{{\left(\sigma_{S}^{AS}\right)}_{c}+{\left(\sigma_{S}^{AS}\right)}_{t}}$$

where ${\left(\sigma_{S}^{AS}\right)}_{c}$ and ${\left(\sigma_{S}^{AS}\right)}_{t}$ denote the start stress of the austenite-to-martensite transformation under compression and tension, respectively. The Nitinol material properties at 37 °C, as summarised in Table 1, were derived from experimental studies on biomedical-grade peripheral Nitinol stents, following the methodology outlined by Petrini et al. [56] and Wu et al. [57].

### 2.2. Design of Stents

Currently, a sequential Z-shaped ring design is common in most commercially available stents. This structure includes two main components: Z-shaped rings, also called “struts,” which undergo expansion during deployment to maintain vessel patency, and connectors or “bridges”, which provide flexibility for navigating the curved paths of arteries [58].

**Figure 4:**
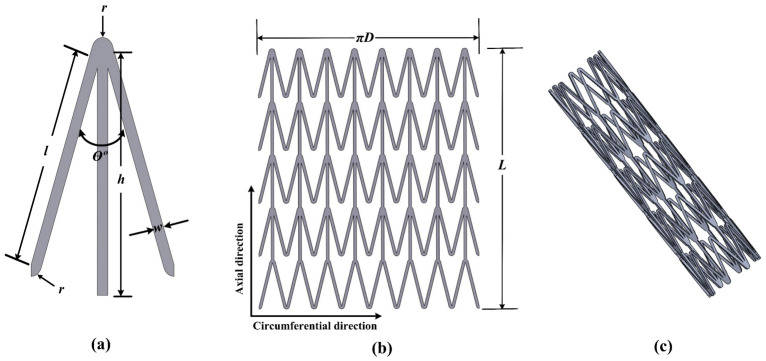
Stent design with arrow-shaped ZPR structure. (**a**) The unit cell, where *l* is the length of the hinge strut; *ϴ* is the hinge angle between these ribs; *r* is the crown radius; *w* is the width of each strut; and *h* is the connector strut height. (**b**) The planar 2D structure, where *D* and *L* represent the stent’s overall diameter and length, respectively. (**c**) 3D tubular form.

To address the issues of longitudinal foreshortening and elongation, we developed a stent based on a zero Poisson’s ratio (ZPR) architecture, inspired by a Z-shaped missing-rib foam structure known for its NPR behaviour [59]. The 3D model of the ZPR repeatable unit, illustrated in Figure 4, was created using SOLIDWORKS 2025 (Dassault Systèmes SOLIDWORKS Corp., Waltham, MA, USA). The stent’s structural unit (Figure 4a) is characterised by key geometric parameters, whereby *l* denotes the length of the hinge strut in the arrow-shaped cell; *ϴ* is the hinge angle between these ribs; *h* represents the connector strut height; *r* is the crown radius; and *w* is the width of each strut. As shown in Figure 4b, the design process begins with a 2D planar pattern, where *D* and *L* represent the stent’s overall diameter and length, respectively. This layout was subsequently wrapped around the outer surface of a cylinder to form the final 3D tubular configuration (Figure 4c).

**Figure 5:**
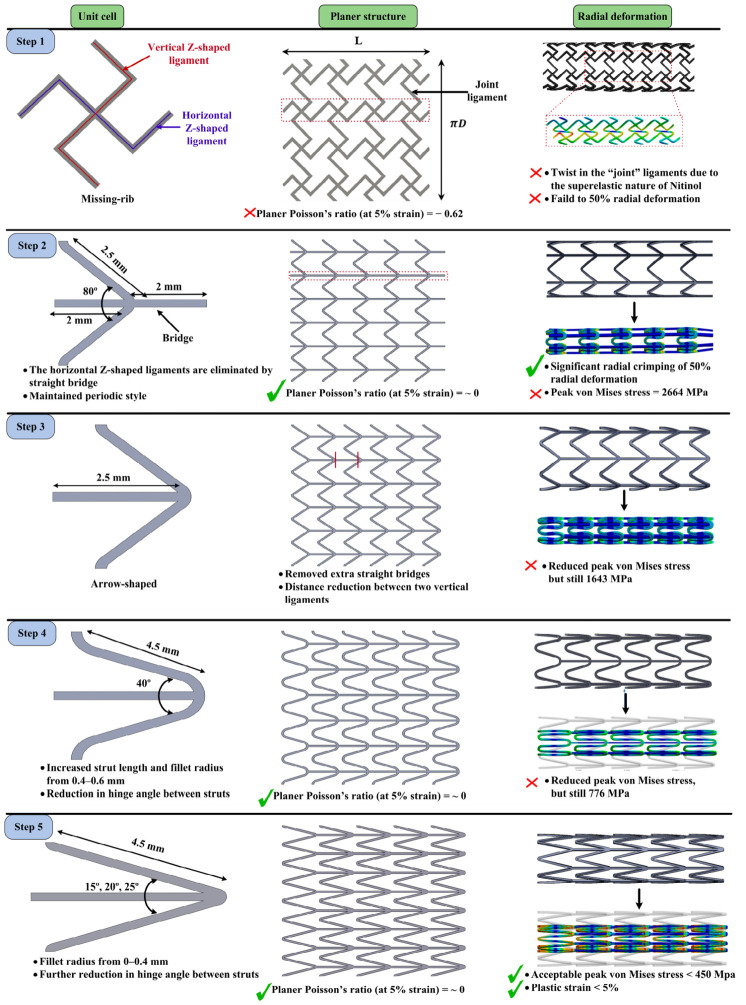
Preliminary design assessment of the ZPR structure. Designs meeting the required criteria are marked with a green check, whereas noncompliant designs are indicated by a red cross.

The missing-rib design is extensively utilised in lightweight, high-stiffness, and flexible mechanical metamaterials, particularly in aerospace and energy-absorbing structures [60,61]. Barletta et al. [62] have demonstrated that these structures have excellent shape recovery, making them environmentally friendly options for impact-absorbing barriers. However, when adapted to a self-expanding Nitinol stent, the original missing-rib layout proved unsuitable for radial crimping. Unlike balloon-expanding stents, which plastically deformed during deployment, self-expanding Nitinol stents must undergo large, fully recoverable deformation during crimping and deployment, making them highly sensitive to geometric instability and stress concentration [48]. As a result, the superelastic properties of Nitinol caused the “joint” ligaments to twist (as shown in Figure 5, Step 1). The relevant radial crimping procedures that were applied are explained further in Section 2.3.

To overcome this, the “horizontal Z-shaped” ligaments were replaced with straight connectors acting as “bridges”. By retaining the “vertical Z-shaped” ligaments and integrating them with these straight bridges (Figure 5, Step 2), we created an arrow-shaped periodic ZPR structure capable of radial deformation. Accordingly, Poisson’s ratio (γ) is calculated by dividing the transverse strain ($\varepsilon_{i}$) by the axial strain ($\varepsilon_{j}$) in the direction of the applied load, as expressed in Equation (2):

(2) $$\gamma =-\frac{\varepsilon_{i}}{\varepsilon_{j}}$$

As shown in Figure 5, a step-by-step preliminary design assessment of the stent ZPR design was conducted to evaluate the structural feasibility, safety, and efficiency of the stent’s ZPR design under significant radial deformation. The primary goal was to determine whether the ZPR structure could withstand deformation while keeping peak von Mises stress within the material’s allowable limit. In Steps 3 and 4, an arrow-shaped ZPR configuration with an approximately zero Poisson’s ratio was obtained; however, the peak stress exceeded the material’s AS finish stress threshold, $\sigma_{F}^{AS}$, indicating an unsafe design due to the increased risk of inelastic deformation and compromised long-term mechanical reliability under cyclic radial compression [63]. Following several design iterations that modified the hinge angle, rib/strut length, and fillet radius, the final design in Step 5 successfully met the stress safety criteria.

### 2.3. Structural FEA Modelling of Radial Crimping

The radial crimping procedure is a widely accepted technique for evaluating the mechanical behaviour of stents [12,64,65], as it replicates the loading conditions encountered during catheter insertion and delivery to the treatment location. In this study, the Static Structural solver in ANSYS Mechanical 2024 R1 (Ansys Inc., Canonsburg, PA, USA) was employed to perform the radial crimping simulation.

#### 2.3.1. Boundary Conditions

**Figure 6:**
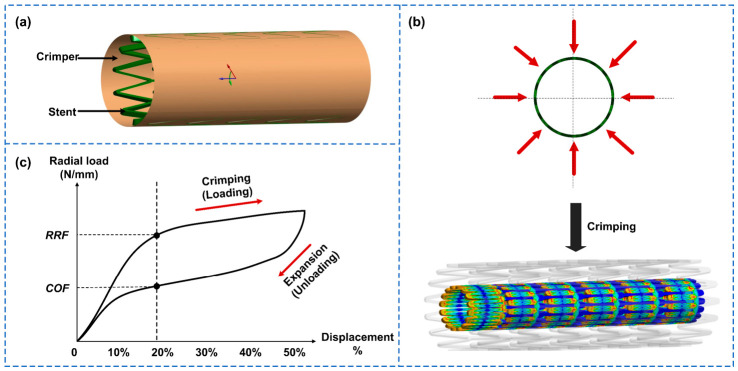
(**a**) Radial crimping model, (**b**) radial compression, and (**c**) RRF and COF are evaluated at the implementation displacement during crimping and expansion.

Simulation of radial crimping was achieved by applying contact with a rigid cylindrical crimping head (Figure 6a). This cylindrical crimper mimicked the radial compression mechanism of experimental setups, typically used following the ASTM F3067-14 standard [66]. The simulation involved a two-step boundary condition: crimping and expansion. A cylindrical coordinate system was used, where the x-axis, y-axis, and z-axis corresponded to the radial, circumferential, and longitudinal directions, respectively. A quarter model of the stent was used in the circumferential direction, with constraints applied on both circumferential ends in the y-direction. Additionally, the longitudinal ends were fully constrained, and the model was fixed in the z-direction at its centre to ensure stability.

During the crimping step, the cylindrical crimper was subjected to a negative radial displacement, reducing the stent’s outer diameter from its initial value of 6.50 to a minimum of 3.25 mm, corresponding to a 50% radial deformation (Figure 6b). This process replicates the stent crimping that is required before insertion into the artery and its navigation through the diseased region. The induced compression stores substantial strain energy within the stent, allowing it to subsequently self-expand. Later, the expansion step involved releasing the stent, permitting it to regain its original diameter. The initial assembly of the stent and crimper was aligned coaxially, with the crimper’s inner diameter set marginally larger than the stent’s outer diameter. A frictionless contact between the stent’s outer surface and the crimper’s inner surface was assumed and modelled using the Augmented Lagrange formulation. To constrain rigid body movement throughout the analysis, a remote displacement constraint was applied to a central node on the stent. The FEA results were subsequently utilised to evaluate the mechanical performance of the stent and were later included in the development of the optimisation framework (Section 2.4.2).

**Radial resistive force (RRF) and chronic outward force (COF):** RRF denotes a compressive force that the stent generates to resist external compression, thereby preserving its structural integrity [16]. COF, in comparison, refers to an expanding force exerted by the stent onto the arterial wall, promoting vessel expansion and maintaining lumen patency. In this context, the stent must exert an adequate COF to support the diseased artery and to prevent collapse under vascular pressure [67]. Self-expanding stents are generally designed larger than the target vessel diameter to achieve adequate lumen expansion and secure placement. In this study, a stent-to-vessel oversizing ratio of 1.20 was assumed. For a stent with an outer diameter of 6.50 mm, this corresponds to a target vessel diameter of 5.42 mm, representing approximately 20% oversizing. This ratio was selected because it falls within the clinically and biomechanically supported range for self-expanding Nitinol peripheral stents. Previous studies indicate that oversizing ratios of 1.10–1.40 can improve lumen gain, reduce incomplete stent apposition, and support favourable fatigue performance [14,68,69], whereas excessive oversizing above 1.40 may increase radial force without further mechanical benefit [70,71]. Accordingly, RRF and COF were extracted from the force–displacement response obtained from the FEA at 20% radial displacement. Specifically, RRF was defined as the reaction force exerted by the stent on the inner crimper surface during the crimping (loading) stage, while COF was defined as the outward reaction force during the expansion (unloading) stage at the same radial displacement (Figure 6c). The overall length of the ZPR stents was 20.5 ± 2 mm.

**Foreshortening (FS):** Near-zero FS reflects improved precision in stent positioning post-expansion at the target location, where this helps minimise vessel friction that could harm the sensitive endothelial lining [47,53]. Although clinically relevant foreshortening is commonly associated with the expansion stage, previous stent optimisation studies have also evaluated FS during radial crimping, particularly at the minimum crimped diameter, to quantify the maximum axial length change under large deformation [65]. Following this approach, FS was evaluated in the present study at 50% radial crimping to assess the axial deformation tendency of the proposed stent designs under the most severe crimping condition. This approach is relevant because axial–radial coupling during crimping provides insight into the geometric stability. A stent that undergoes substantial axial elongation or shortening during crimping may experience the opposite axial recovery during expansion, which can influence deployment accuracy [14]. In the present computational framework, the stent was not deployed inside a deformable artery and was allowed to recover freely during unloading using an ideal superelastic Nitinol material model; therefore, the final axial length change after expansion was negligible. Accordingly, FS (%) was determined as $\frac{L_{0}-L_{final}}{L_{0}}\times 100$, where $L_{final}$ and $L_{0}$ represent the length following 50% radial crimping and the initial stent length, respectively. These lengths were measured by extracting the axial displacement of the stent’s end faces before and after crimping, using probe tools and geometry coordinates from the deformed shape.

**Peak stress (PS) or von Mises stress:** PS is a key indicator of the structural integrity of Nitinol stents. For safety assurance, its maximum value was restricted to the Nitinol’s AS finish stress threshold, $\sigma_{F}^{AS}$, as listed in Table 1, based on the superelastic stress–strain behaviour shown in Figure 3. The PS was evaluated at 50% radial displacement, representing the maximum stress experienced during the crimping process.

#### 2.3.2. Meshing

**Figure 7:**
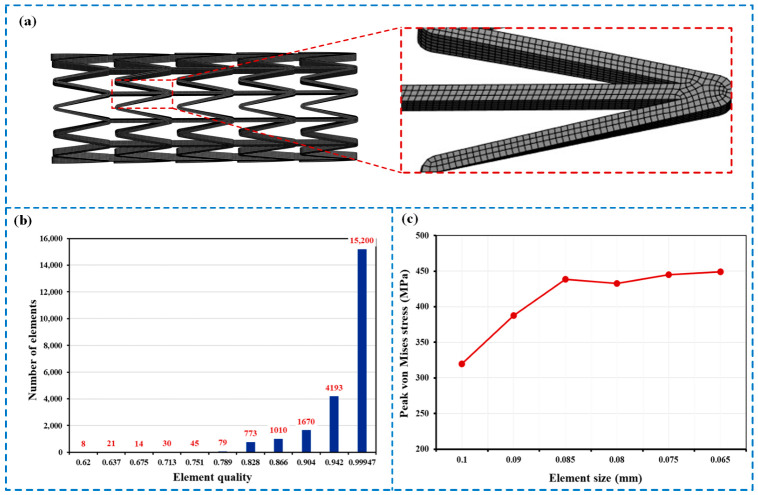
Mesh study of FE simulation. (**a**) Hexahedral elements via sweep meshing. (**b**) Element quality of the mesh. (**c**) Convergence of the mesh.

A high-quality, structured mesh was generated using 3D solid hexahedral elements via sweep meshing (Figure 7a), which is well-suited to the tubular geometry of vascular stents. Hexahedral elements were selected over tetrahedral elements due to their superior numerical performance in capturing bending behaviour, contact interactions, and material nonlinearity [72,73]. The mesh was configured for nonlinear mechanical analysis with linear element order, utilising SOLID185 elements that are suitable for large deformations and nonlinear material properties. A globally uniform mesh was adopted to maintain consistency and reduce meshing complexity.

A mesh convergence study was conducted by progressively reducing the element size from 0.1 mm to 0.065 mm while monitoring the peak von Mises stress. According to Figure 7c, the stress values began to stabilise from an element size of 0.085 mm onward, with variations between successive refinements falling below 2%. The peak von Mises stress converged near 450 MPa, confirming mesh independence. Based on this, an efficient mesh was achieved with element sizes varying between 0.085 mm and 0.75 mm, where a total number of elements between 23,000 and 38,500 occurred, depending on the design. Additionally, Figure 7b shows that the majority (over 95%) of mesh elements scored above 0.60 in quality, confirming the robustness of the simulation results [36,74].

### 2.4. Optimisation Procedure

#### 2.4.1. Response Surface Methodology (RSM)

RSM combines statistical techniques and mathematical modelling to analyse and optimise the relationship between multiple input factors or variables and desired outcomes. In this study, RSM was employed alongside the Design of Experiments (DoE), enabling efficient exploration of the combined effects of various design parameters on performance metrics, allowing for optimisation with a minimal number of strategically planned simulations. DoE maximises the extraction of relevant information with fewer trials, while RSM identifies ideal configurations for achieving target responses.

Compared to other optimisation approaches, such as the Taguchi method, genetic algorithms, or surrogate models [75,76], RSM is favoured due to its balanced accuracy and its generation of unbiased predictive models [77]. Further, it was utilised here to evaluate interactions among design variables and their effects on key mechanical behaviours. By applying a second-order polynomial regression model via the least-squares method, RSM predicts optimal parameter combinations. The generalised quadratic model representing system behaviour [78] is

(3) $$y=\beta_{0}+\sum_{i=1}^{k}\beta_{i}x_{i}+\sum_{i=1}^{k}\beta_{ii}x_{i}^{2}+\sum_{i<j}^{k}\beta_{ij}x_{i}x_{j}+\varepsilon$$

where *y* is the predicted output, *k* is the number of input variables; *x_i_* and *x_j_* are the *i*-th and *j*-th variables, respectively; *β*_0_ is the intercept; *β_i_* is the linear coefficient; *β_ii_* is the quadratic coefficient; *β_ij_* is the interaction coefficient; and *ε* is the error between statistical and FEA results. The second-order polynomial model was utilised as it is capable of capturing both linear effects and nonlinear interactions between geometric parameters [77,78]. Analysis of variance (ANOVA) for RSM analysis was carried out via MINITAB 22 (Minitab, LLC, Chicago, IL, USA) to identify the effects of input design variables on mechanical behaviour at a significance level of 0.05 (95% confidence).

#### 2.4.2. Design of Experiments (DoE)

The primary goal was to enhance self-expanding ZPR stent performance by maximising beneficial responses such as COF and RRF, while simultaneously minimising undesired effects like FS, and ensuring PS remained within safe limits, specifically below the austenite finish yield stress, $\sigma_{F}^{AS}$. Therefore, a total of four performance responses were measured by varying three key design factors (hinge angle, strut thickness, and strut length), where each was analysed at three levels (high: +1, medium: 0, low: −1) in this research. The ranges for the high and low levels of design parameters (Table 2) were determined through the preliminary design evaluation described in Section 2.2, whilst also taking into account the constraints imposed by the µ-3D printing process used for fabricating Nitinol stents [42,43,44].

The selected design parameters (*ϴ*, *t*, and *l*) in Table 2 were chosen due to their direct effect on the deformation mechanism of the ZPR structure. Specifically, the *ϴ* governs the rotational behaviour of unit cells and the axial–radial coupling behaviour, the *t* controls bending stiffness and load-bearing capacity, and *l* affects structural flexibility and deformation compatibility during radial crimping and expansion.

A central composite design (CCD) strategy [78] for DoE was employed to efficiently capture both the curvature of the response surface and potential nonlinear interactions among design variables, resulting in 15 finite element (FE) simulations of the radial crimping process (Table 3). This is particularly essential for stent structures, where mechanical responses are governed by nonlinear deformation mechanisms, such as strut bending, rotation, and surface-to-surface contact. The total number of design points, *N*, was determined using Equation (4) [78]:

(4) $$2^{k}+2k+n_{0}=N$$

where the number of design parameters, *k*, is 3, and the number of centre points, *n*_0_, is 1. The objective and constraint functions were evaluated via FEA, and the resulting data were analysed using RSM. Within the RSM framework, the second-order polynomial regression model (Equation (3)) was employed to approximate the connection between design variables and the optimisation goals (Equation (5)) and non-linear constraints (Equation (6)). Thus, the optimisation problem can be formulated as follows:

(5) $$\{\begin{array}{c} \varphi_{max}=COF\left(∆\right)\\ \varphi_{max}=RRF\left(∆\right)\\ \varphi_{min}=FS\;\left(∆\right) \end{array}$$

The optimisation objectives were formulated to reflect clinically relevant performance requirements, where higher RRF and COF correspond to improved vessel scaffolding capability, while minimising FS (or axial elongation) ensures deployment accuracy and dimensional stability.

Subject to

(6) $$\{\begin{array}{c} PS\left(∆\right)\;˂\;\sigma_{F}^{AS}\;\;\;(structural\;safety)\\ -0.010\leq FS\;\left(∆\right)\leq \;0.010\;\;\;(dimentional\;stability) \end{array}$$

These constraints ensure that the stent functions within the superelastic regime of Nitinol and maintains axial deformation within ±1% of its original length. From a practical standpoint, the crimping force (peak radial force) required to achieve the target radial deformation should remain within a feasible range to ensure deliverability. Although this constraint was not explicitly included in the optimisation formulation, excessively high stiffness and the associated crimping force may restrict large deformation during crimping and adversely affect practical deployment.

(7) $$∆={\left\{ϴ,t,l\right\}}^{T}\;\;\;\;\;\;where\;\{\begin{array}{c} 15{}^{\circ}\leq ϴ\leq 25{}^{\circ}\\ 0.20\leq t\leq 0.50\\ 4.50\leq l\leq 6.00 \end{array}$$

It should be noted that the RSM framework captures global trends within the defined design space; however, it does not directly capture local deformation phenomena such as strut-to-strut contact and instability. These effects are therefore analysed separately through detailed FE simulations.

## 3. Results and Discussion

Computational modelling tools play a critical role in shaping and advancing vascular stent designs, minimising the need for extensive prototyping and experimental trials, thereby reducing both development time and associated costs [79]. Moreover, computer modelling and simulation allow for the refinement of stent designs by enabling a structured and efficient development process. This leads to devices with enhanced mechanical performance and the potential for better clinical outcomes, effectively addressing the shortcomings of conventional trial-and-error methods. While ZPR architectures represent a novel and promising approach, their computational evaluation has remained limited for 3D-printed Nitinol stents [53,80]. This research introduces, for the first time, a dedicated optimisation framework for ZPR-based stents, aiming to improve their structural performance and clinical utility in treating PAD.

After the training and testing phases, ANOVA was conducted to evaluate how input design parameters (i.e., hinge angle, strut thickness, and strut length) influence mechanical performance and to determine the relative importance of each factor. Four key performance metrics (i.e., RRF, COF, FS, and PS) were assessed across all 15 design points, as presented in Table 3. During the measurement of the axial dimension after radial crimping of the ZPR stent, slight axial shortening was detected, as indicated by small negative values of foreshortening. However, the magnitude was close to zero.

### 3.1. Evaluation of Model Adequacy

**Figure 8:**
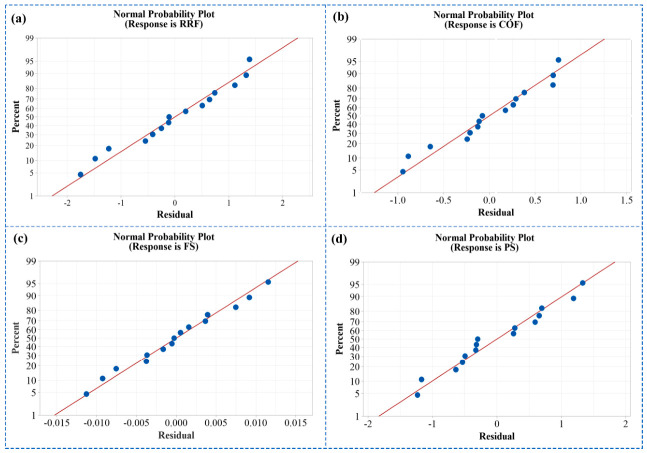
Normal probability plots that display the residuals associated with (**a**) RRF, (**b**) COF, (**c**) FS, and (**d**) PS.

The quality of the regression model is illustrated in Figure 8, where the predicted values of performance metrics obtained from the statistical model (Equation (3)) are compared against the actual results derived from finite element simulations. The close alignment of residuals in both the scatter plot and the standard probability plot supports the validity of the normality assumption. A strong linear correlation was observed for RRF, COF, FS, and PS, with corresponding R-squared (R^2^) values of 94.31%, 94.82%, 91.53%, and 89.65%, respectively, indicating high predictive accuracy of the developed statistical models.

### 3.2. Effect of the ZPR Design Parameters

As previously discussed, stent design plays a critical role in the treatment of PAD. Two essential characteristics of this are as follows: radial stiffness, which ensures the vessel remains open; and flexibility, which facilitates smooth navigation and precise placement during delivery [53]. A balanced outward force is also vital for effectively expanding calcified plaques [28]. Additionally, achieving minimal foreshortening or elongation after deployment enhances placement accuracy and reduces friction against the vessel wall, protecting the delicate endothelial lining [47]. The dimensions and geometry of the unit cell largely influence these performance traits. To investigate how ZPR unit cell design parameters affect RRF, COF, FS, and PS, an ANOVA was conducted using full quadratic terms. The ANOVA results revealed that all three design parameters (i.e., hinge angle, *ϴ*; strut length, *l*; and strut thickness, *t*) had a statistically significant impact on the response variable (*p* < 0.05), demonstrating their strong influence on the performance metrics of RRF, COF, FS, and PS.

**Figure 9:**
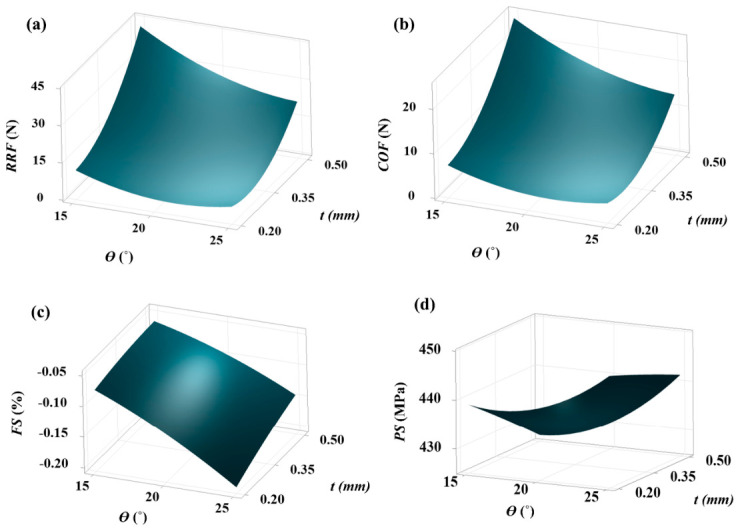
3D response surfaces illustrating the joint effects of hinge angle and strut thickness on response variables, i.e., (**a**) RRF, (**b**) COF, (**c**) FS, and (**d**) PS.

**Figure 10:**
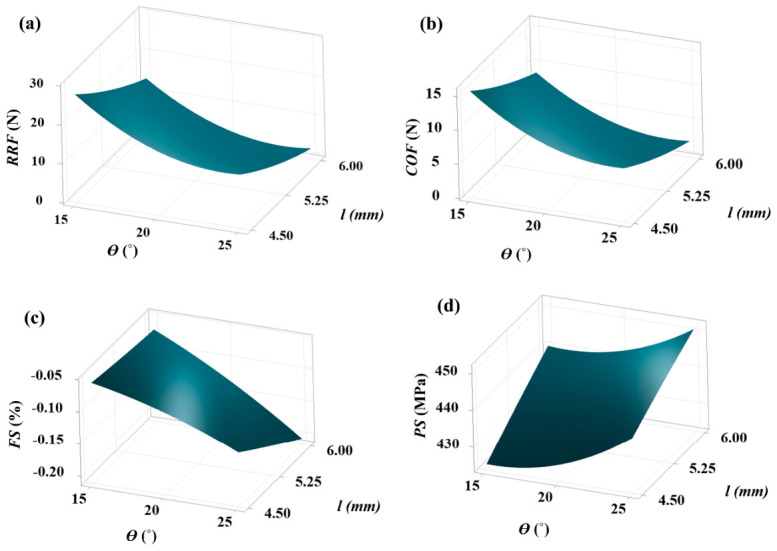
3D response surfaces illustrating the joint effects of strut length and hinge angle on response variables, i.e., (**a**) RRF, (**b**) COF, (**c**) FS, and (**d**) PS.

**Figure 11:**
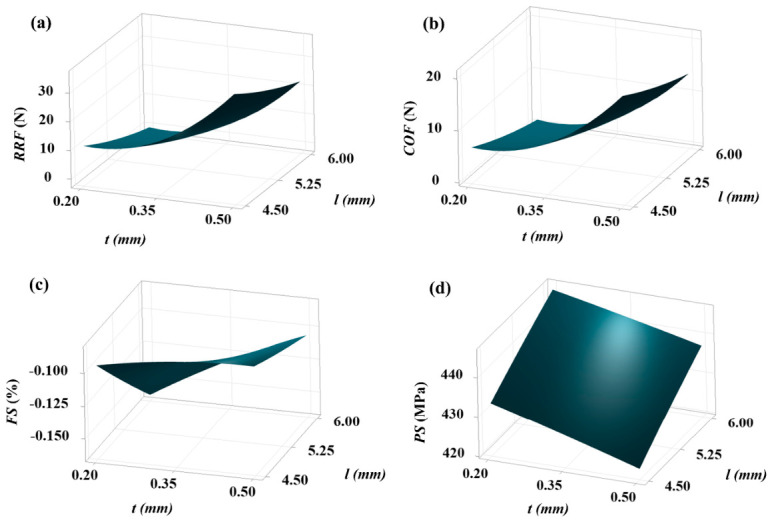
3D response surfaces illustrating the joint effects of strut thickness and strut length on response variables, i.e., (**a**) RRF, (**b**) COF, (**c**) FS, and (**d**) PS.

The 3D response surface analysis, including two-way interaction effects shown in Figure 9, Figure 10 and Figure 11, illustrated how pairs of design parameters (*ϴ*, *t*, or *l*) jointly influence each evaluated response (RRF, COF, FS, and PS). Two dependent design parameters were plotted alongside each response surface’s performance metric, while the remaining independent parameter was kept constant at its baseline (midpoint) value. Accordingly, it was observed that both the stent’s resistance to radial compression and the outward radial force applied to reopen the artery increased with decreasing hinge angle (*ϴ*) and the strut length (*l*), and with increasing strut thickness (*t*) (Figure 9a,b, Figure 10a,b and Figure 11a,b). These trends are consistent with those reported in previous studies [28,55,81]. In particular, at a lower hinge angle (15°), both RRF and COF exhibit a more pronounced sensitivity to variations in strut thickness (Figure 9a,b) and length (Figure 10a,b). For instance, increasing the strut thickness from 0.20 mm to 0.50 mm results in a substantial increase in RRF (approximately 12 N to 45 N), while reducing the strut length from 6.00 mm to 4.50 mm increases RRF from approximately 15 N to 28 N. This behaviour can be attributed to the geometric reconfiguration of the unit cell, where decreasing *ϴ* and increasing *t* promote a transition from an axially oriented rhombic structure to a more radially aligned configuration, thereby enhancing structural stiffness and radial load-bearing capacity. Conversely, larger hinge angles and longer strut lengths produce a more open-cell geometry, resulting in increased flexibility and reduced radial stiffness. Notably, at a higher strut thickness (*t* = 0.50 mm), the influence of hinge angle becomes more significant, with both RRF and COF increasing sharply as *ϴ* decreases (Figure 9a,b). This increase in radial strength results in a reduction in radial recoil [18].

In terms of axial stability, near-zero negative FS values indicate slight axial elongation after deployment, corresponding to a minimal increase in stent length relative to its initial configuration. FS was strongly affected by *ϴ*, with decreasing *ϴ* shifting the negative FS values closer to zero, likely due to the dominant role of *ϴ* in dictating stent motion during radial compression [65]. As shown in Figure 9c, Figure 10c and Figure 11c, the axial elongation increased with increasing hinge angle and strut length but decreased with increasing strut thickness. At *ϴ* = 25°, the elongation increases noticeably (from approximately −0.15% to −0.20%) as strut thickness decreases (Figure 9c). However, this increase is less pronounced at a shorter strut length (*l* = 4.50 mm) compared to a longer length (*l* = 6.00 mm), indicating a weaker sensitivity of elongation to thickness at lower strut lengths (Figure 11c).

Lastly, Figure 9d, Figure 10d and Figure 11d illustrate that PS decreases with increasing *t* (e.g., from 430.95 MPa to 418.80 MPa) but rises sharply with increasing *ϴ* and *l*. When both the hinge angle and strut length increase simultaneously, the stent behaves more like a flexible spring. However, this increased flexibility results in intensified bending moments and curvatures, especially at hinge regions, thereby elevating peak stress [5]. In contrast, thicker struts, due to their larger cross-sectional area, are better able to distribute applied loads and reduce localised deformation, thus minimising stress concentrations [82].

### 3.3. Optimisation

According to Equations (5)–(7), the RSM, employed as a multi-objective optimisation approach, identified an optimal stent design at a hinge angle of 15°, a strut thickness of 0.5 mm, and a strut length of 4.50 mm. This design yielded the highest RRF and COF while minimising foreshortening or elongation. The optimisation was carried out under simulated radial crimping conditions, accurately capturing the stent’s mechanical behaviour during deployment by evaluating RRF, COF, and FS. The optimised stent exhibited RRF and COF values of 51.07 N and 29.03 N, respectively, along with a negligible foreshortening of −0.056%. These values are comparable to those reported for several commercial self-expanding Nitinol stents used in PAD interventions, such as the *Acculink™ (Abbott)*, *Cristallo Ideale*, *s.m.a.r.t* and *Protégé* stents [83,84,85]. In contrast, conventional stents typically exhibit a shortening rate in the range of 3–5% [53]. This confirms that the proposed geometry provides excellent axial stability upon expansion. As a result, the design is expected to deliver enhanced radial strength and chronic outward force, which are critical for effective vessel scaffolding and long-term patency. Moreover, the extremely low foreshortening indicates improved deployment precision compared to most commercial counterparts.

To validate the accuracy of the RSM-based regression model, predicted responses were compared with actual values obtained from FE simulations. This comparison revealed a very low prediction error ranging from 0.60% to 5.71% (Table 4), confirming the strong consistency and reliability of the developed model. Thus, this demonstrates a strong concordance between the simulation outcomes and the predicted results.

### 3.4. Comparative Analysis Under Radial Compression

#### 3.4.1. Comparison of Optimised ZPR Structure with PPR and NPR Stents

**Figure 12:**
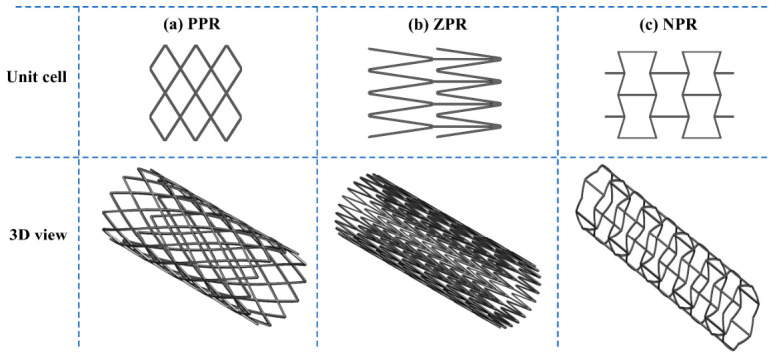
Stent architectures used for comparative analysis under radial compression: (**a**) PPR—a conventional zig-zag pattern, adapted from [52]; (**b**) ZPR—an arrow-shaped geometry; and (**c**) NPR—a re-entrant auxetic structure, adapted from [52].

To evaluate the performance of our proposed ZPR structural stent, two additional configurations were analysed for comparison: a commercially available zig-zag structure exhibiting PPR behaviour, such as *WALLSTENT^TM^*, and a re-entrant structure widely used in research, known for its NPR characteristics (Figure 12). However, research involving Nitinol-based self-expanding stents with both these PPR and NPR structures remains limited, particularly with regard to radial force and foreshortening characteristics [81,86,87]. Therefore, for consistency and validity, our comparisons were based on the stent geometry, material, and methodological framework reported by Prithipaul et al. [52]. The material modelled in this case was elastoplastic, characterised by an elastic modulus *E* of 193 GPa, a Poisson ratio *ν* of 0.3, yield strength *σ_y_* of 260 MPa, and tangential modulus *E_t_* of 0 GPa.

Table 5 displays the corresponding results. The proposed structure demonstrated enhanced radial stiffness compared to the PPR structure while exhibiting moderately lower stiffness relative to the NPR structure. This suggests a balanced radial support, avoiding both excessive rigidity and inadequate mechanical support for arterial applications. Furthermore, the proposed design achieved near-zero foreshortening, in contrast to both PPR and NPR configurations, indicating improved deployment accuracy and a more stable, frictionless interface with the arterial wall.

**Figure 13:**
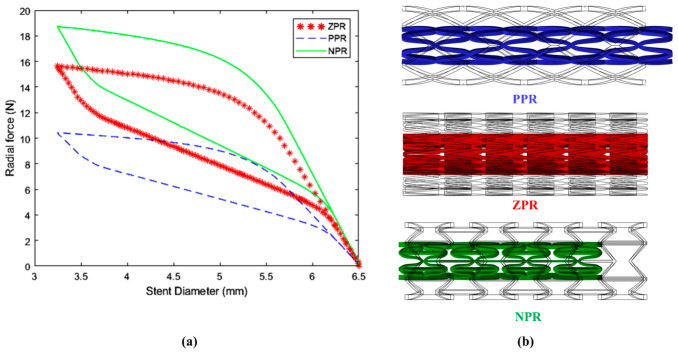
Comparison between Nitinol self-expanding PPR, ZPR, and NPR structural stents in terms of (**a**) radial force, and (**b**) foreshortening.

In the study, the PPR and NPR stent structures were re-simulated using Nitinol, following the process detailed in Section 2.3, to assess the mechanical characteristics of the optimised ZPR structural stent. As shown in Figure 13a, the PPR structure generated a substantially lower radial force compared to the ZPR stent. This is attributed to the axial shortening that occurs during radial expansion in PPR designs, which reduces radial stiffness and contributes to foreshortening (Figure 13b) [65]. Conversely, the NPR stent demonstrated the maximum radial force among the three configurations, though only marginally greater than the ZPR structure. This enhancement is due to axial elongation during radial expansion, which increases the contact area and vessel support. However, this elongation may complicate deployment in tortuous anatomies by reducing positional accuracy [47]. Numerically, the optimised ZPR stent demonstrated approximately 50% greater radial force compared to the PPR design, while showing a 16.67% reduction relative to the NPR counterpart. It also maintained excellent axial stability, with negligible post-deployment foreshortening or elongation (|FS| < 1%). By preserving its axial length during expansion, the ZPR stent reduces stress on the vessel wall and offers a balanced, stable RRF and COF.

#### 3.4.2. Comparison Among ZPR Configurations

While the above comparison demonstrates the overall advantages of the self-expanding ZPR stent concept over conventional PPR and NPR designs, further insight into the influence of geometric parameters within the ZPR configurations is required. A targeted comparison was conducted on selected ZPR configurations with fixed strut thickness (*t* = 0.50 mm) and strut length (*l* = 4.50 mm), chosen based on practical manufacturing considerations and consistency with the fabricated samples. The corresponding experimental results are discussed in Section 3.5. The hinge angle, ϴ, was varied (15°, 20°, 25°, and 30°) to evaluate its influence on structural response under radial compression. It should be noted that the 30° configuration lies outside the selected design space defined for the parametric study. However, it is included here to provide a broader perspective on deformation characteristics and to illustrate the influence of hinge angle beyond the optimised range.

**Figure 14:**
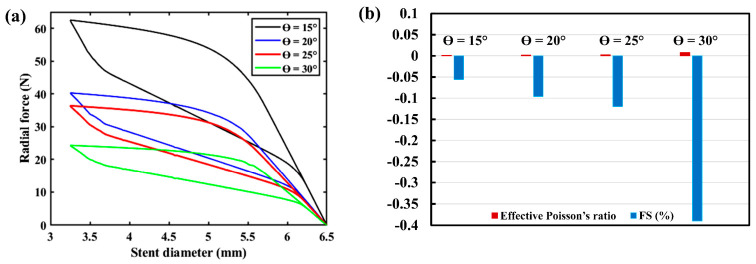
Comparison of ZPR stents with varying hinge angles (ϴ = 15°, 20°, 25°, and 30°) under radial compression: (**a**) radial force-diameter response; (**b**) foreshortening (FS) and effective Poisson’s ratio ($v_{eff}$) for the corresponding configurations.

**Figure 15:**
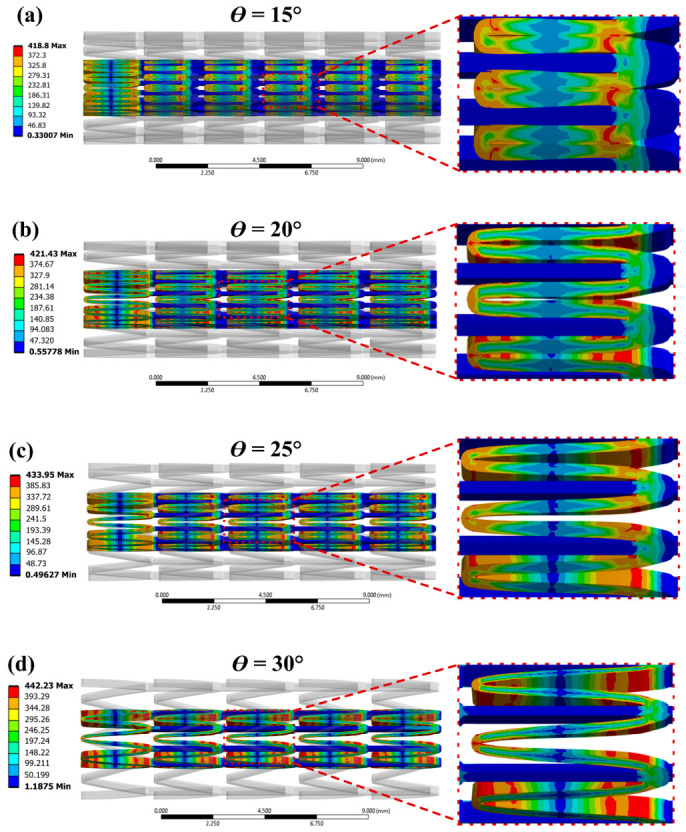
Deformation behaviour and von Mises stress distribution of ZPR stents with different hinge angles under radial compression: (**a**) ϴ = 15°, (**b**) ϴ = 20°, (**c**) ϴ = 25°, and (**d**) ϴ = 30°. The contour plots illustrate the evolution of stress distribution and deformation patterns, with magnified views highlighting local behaviour at the strut regions.

As shown in Figure 14a, the 15° configuration exhibits the highest peak radial force (62.55 N) and the steepest initial slope, indicating a stiffness-dominated response. With increasing hinge angle, both radial stiffness and peak force decrease progressively, and the 30° configuration demonstrates the most compliant behaviour with the lowest peak force of 24.32 N. This trend correlates directly with the deformation patterns presented in Figure 15. The 15° ZPR stent undergoes limited geometric deformation (Figure 15a), resulting in higher structural resistance and elevated force output, whereas higher hinge-angle configurations permit increased geometric flexibility.

Despite producing the highest radial force, the 15° configuration shows comparatively lower and more uniformly distributed von Mises stress. In contrast, the 30° configuration exhibits pronounced localised stress concentrations (Figure 15d), particularly along the strut width and at hinge and fillet regions, even under lower global force. This indicates a bending-dominated deformation mode, where stress is concentrated locally rather than distributed across the structure [5]. Furthermore, the deformation patterns (Figure 15a,b) reveal that lower hinge-angle configurations (15° and 20°) experience early strut-to-strut interaction during crimping, leading to the restriction of large deformation during crimping. In contrast, the 25° configuration shows delayed or negligible strut interaction, resulting in a smoother and more uniform deformation process.

The axial deformation and effective Poisson’s ratio results (Figure 14b) substantiate these observations. While the Poisson’s ratio of the planar lattice structure was evaluated under uniaxial tensile loading to characterise the intrinsic deformation behaviour of the ZPR unit cell in Section 2.2, the effective Poisson’s ratio of the stent, $v_{eff}$, is calculated here under radial compression, representing the global structural response of the tubular geometry, as defined in Equation (8).

(8) $$v_{eff}=-\frac{\varepsilon_{axial}}{\varepsilon_{radial}}$$

where $\varepsilon_{axial}=(L_{final}-L_{0})/L_{0}$ is the axial strain, with $L_{final}$ and $L_{0}$ representing the deformed and initial stent lengths, respectively, and $\varepsilon_{radial}=(D_{final}-D_{0})/D_{0}$ is the radial strain, with $D_{final}$ and $D_{0}$ denoting the deformed and initial stent diameters. All configurations exhibit negative foreshortening values, indicating slight axial elongation during radial compression. However, the magnitudes of both foreshortening (|FS| ≤ 0.39%) and effective Poisson’s ratio (|$v_{eff}$| ≤ 0.0078) remain very small, confirming minimal axial–radial coupling, as evidenced by the comparison between undeformed and crimped axial lengths in Figure 15. In particular, the 15–25° configurations show extremely low axial deformation (|FS| ≤ 0.12%) and near-zero values of $v_{eff}$ (≤0.0024), confirming near-zero Poisson’s ratio (ZPR) behaviour. This finding represents the lowest reported longitudinal foreshortening in self-expanding stents, to the best of our knowledge. In contrast, the 30° configuration shows a significantly higher foreshortening (0.39%) and a larger deviation in $v_{eff}$, indicating the onset of deviation from ideal ZPR behaviour. Therefore, it is demonstrated that the ZPR behaviour is strongly geometry-dependent and is not preserved beyond a critical hinge angle.

Overall, these results demonstrate that while lower hinge angles (15° and 20°) maximise radial force, they also promote stiffness-dominated behaviour and contact-induced constraints. Although the RSM-based optimisation identified the 15° configuration as the numerical optimum due to its higher radial force, this result does not fully account for deformation stability and practical deployability. Higher hinge angles improve geometric flexibility; however, excessive flexibility, as observed for the 30° configuration, leads to bending-dominated deformation and increased stress localisation along the strut span. In contrast, the 25° hinge angle is considered the most favourable design under the fixed geometric configuration (*t* = 0.50 mm, *l* = 4.50 mm), as it provides a balanced mechanical response, characterised by moderate radial force, reduced stress concentration, minimal strut interaction, and preservation of near-zero Poisson’s ratio behaviour.

### 3.5. µ-3D Printing of ZPR Nitinol Stents

**Figure 16:**
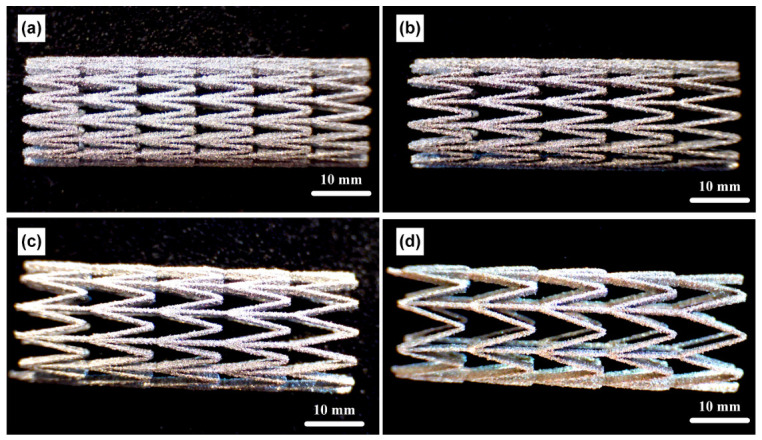
As-printed stents (digital microscope view), designed with a strut length of 4.50 mm, a strut thickness of 0.5 mm, and different hinge angles, such as (**a**) ϴ = 15°; (**b**) ϴ = 20°; (**c**) ϴ = 25°; and (**d**) ϴ = 30°.

Although this study is primarily based on finite element simulations, representative stent designs were manufactured using laser powder bed fusion (PBF-LB) using NiTi (Ni_50.75_ Ti_49.25_, at.%) to provide preliminary physical validation of the simulated designs. The samples were fabricated using a Concept Laser Mlab system under an Ar atmosphere, with a laser power of 70 W, a scan speed of 120 mm/s, a layer thickness of 30 µm, a laser spot size of 40 µm, and a scan rotation angle of 67°, following the processing parameters reported by Lu et al. [88]. While the previous sections focused on the numerical modelling and optimisation of stent geometries (e.g., ZPR), this part shows how those simulated structures were translated into actual-sized prototypes using additive manufacturing. It bridges the gap between simulation and practical feasibility, reinforcing the applicability of the proposed designs. 3D printing facilitates the production of intricate, patient-specific Nitinol vascular stents with optimised geometries that are challenging to produce through conventional fabrication techniques [42,43,45]. The printed samples closely reflect the simulated geometries (Figure 16); however, post-fabrication characterisation revealed deviations in both material properties and structural fidelity.

**Figure 17:**
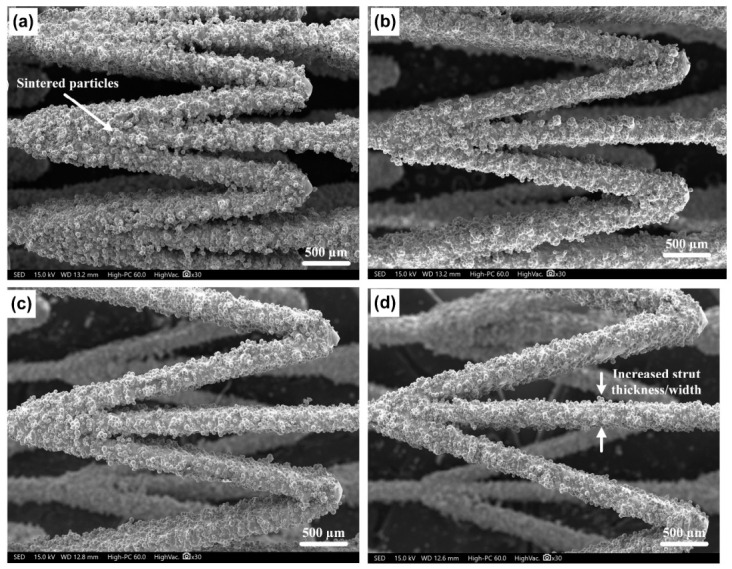
SEM microstructural images of as-printed stent samples, different hinge angles, such as (**a**) ϴ = 15°; (**b**) ϴ = 20°; (**c**) ϴ = 25°; and (**d**) ϴ = 30°.

**Figure 18:**
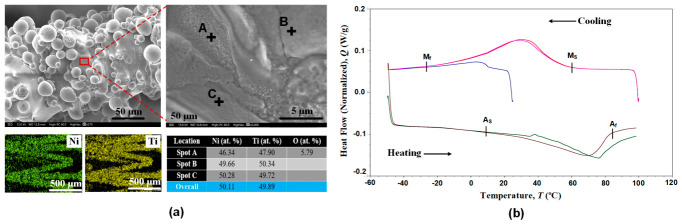
(**a**) SEM–EDS elemental analysis of the as-printed ZPR stent sample, showing the analysed SEM region, Ni and Ti elemental maps, and local composition at selected spots; (**b**) DSC results showing an A_f_ above body temperature.

As a result of the rapid melting and solidification inherent to the PBF-LB process, SEM analysis (Figure 17) revealed microstructural defects, including partially sintered particles and a noticeable staircase effect along the struts, resulting in increased strut thickness relative to the original CAD design [89,90]. Additionally, SEM-based energy-dispersive spectroscopy (EDS) and compositional analysis indicated significant local compositional inhomogeneity (Figure 18a). This microsegregation contributed to a significant shift in phase transformation behaviour [44], as confirmed by DSC (Figure 18b), which showed an elevated austenite finish temperature (A_f_), well above body temperature (37 °C). This indicates that the as-fabricated stents are in a martensitic state at physiological conditions, limiting their ability to demonstrate superelastic behaviour [91].

**Figure 19:**
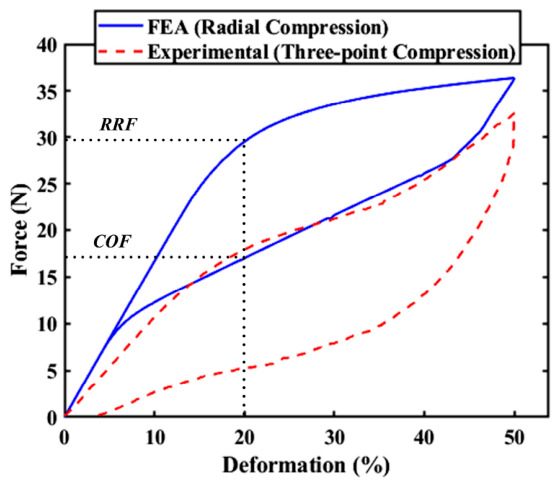
Comparison of experimental (Yasmin et al. [90]) and FEA simulation results of a self-expanding Nitinol stent with the following ZPR design characteristics: *ϴ* = 25°, *t* = 0.50 mm, and *l* = 4.50 mm.

These observations highlight the importance of post-processing treatments to restore functional performance [42,43]. In the previous study by Yasmin et al. [90], appropriate heat-treatment strategies were applied to recover superelastic behaviour and achieve transformation temperatures close to physiological conditions. To provide supporting evidence for the present numerical framework, Figure 19 presents a comparison between the FEA predictions and the experimentally obtained force–displacement response reported in [90], together with the corresponding RRF and COF values derived from both approaches for a representative self-expanding Nitinol stent. The experimental results were obtained using a three-point compression test, which provides a localised approximation of circumferential loading. ASTM F3067-14 specifies that radial loading be applied circumferentially, typically at least three evenly spaced points along the stent length. However, the numerical model assumes ideal radial compression and superelastic material behaviour, whereas the experimental samples exhibit modified material properties and are subjected to different loading conditions. Consequently, this comparison should be interpreted as a preliminary mechanical verification rather than a direct one-to-one validation of the numerical model. Despite these differences, a reasonable agreement in peak force (36.41 N in FE vs. 32.64 N experimentally) confirms that the proposed ZPR geometry captures the overall load-bearing behaviour of the stent. However, the deviations in stiffness and unloading response are attributed to differences in loading conditions, as well as manufacturing-induced variations in geometry and material properties. Full experimental validation under radial compression will be addressed in future work.

## 4. Conclusions

This study investigated the mechanical performance of a proposed arrow-shaped ZPR structural design for self-expanding Nitinol stents, with particular emphasis on RRF, COF, and axial dimensional stability under large-deformation conditions. The main findings are summarised below:

- The influence of geometric design parameters on RRF, COF, and FS was systematically analysed. Both RRF and COF were found to decrease with increasing hinge angle and strut length and to increase with increasing strut thickness. In contrast, lower hinge angles and shorter strut lengths promoted near-zero foreshortening. Notably, the highest RRF (51.22 N) and COF (28.97 N), together with minimal axial deformation (FS = −0.056%), were observed at the minimum hinge angle of 15° and strut length of 4.50 mm, and maximum strut thickness of 0.50 mm.
- A combined computational framework incorporating FEA and RSM was developed to optimise the stent design while considering additive manufacturing constraints. The optimised ZPR configuration demonstrated significantly improved mechanical performance compared to conventional PPR and NPR structures. Specifically, the ZPR stent exhibited approximately 50% higher radial strength than the PPR design; however, it was 16.67% lower than the NPR counterpart and maintained balanced RRF and COF along with near-zero axial deformation (|FS| < 1%).
- Although the RSM-based optimisation identified the 15° hinge angle configuration as the numerical optimum, detailed deformation and stress analyses indicate that a 25° hinge angle provides a more favourable balance between radial strength, structural deformability, and stress distribution. This configuration reduces strut-to-strut interaction and stress concentration while preserving near-zero Poisson’s ratio behaviour. Furthermore, the experimental results show reasonable agreement with FEA predictions, capturing the overall load-bearing behaviour of the self-expanding ZPR stent.

In summary, the proposed ZPR stent demonstrates the capability to achieve near-zero axial deformation while maintaining adequate radial support, highlighting its potential for PAD applications where precise deployment and stable scaffolding are required. While the present study confirms the structural and mechanical feasibility of additively manufactured Nitinol ZPR stents, further experimental validation under realistic radial loading conditions is necessary. In particular, future work should focus on stent–artery interaction, including deployment behaviour, dog-boning, and clinically relevant recoil.

## Figures and Tables

**Table 1:** Properties of Nitinol.

Properties	Symbol	Values
Isothermal temperature (°C)	*T*	37
Poisson’s ratio	*ν*	0.30
Young’s modulus—Austenite (MPa)	*E_a_*	51,100
Young’s modulus—Martensite (MPa)	*E_m_*	51,100
Start stress of AS transformation under loading (MPa)	$\sigma_{S}^{AS}$	416
Finish stress of AS transformation under loading (MPa)	$\sigma_{F}^{AS}$	450
Start stress of SA transformation under unloading (MPa)	$\sigma_{S}^{SA}$	185
Finish stress of SA transformation under unloading (MPa)	$\sigma_{F}^{SA}$	104
Transformation strain (mm/mm)	$\varepsilon_{L}$	0.05
Tension and compression behaviour difference	*α*	0.19

**Table 2:** Design parameters of the ZPR structure.

				Levels		
Design Parameters		Annotation	Unit	−1	0	+1
Variable	Hinge angle between the struts	*ϴ*	(°)	15.00	20.00	25.00
	Strut thickness	*t*	mm	0.20	0.35	0.50
	Hinge strut length	*l*	mm	4.50	5.25	6.00
Constant	Strut width	*w*	mm	0.20		
	Connector strut height	*h*	mm	5.50		
	Crown radius	*r*	mm	0.40		

**Table 3:** ZPR design parameters and respective performance responses.

No.	Input Design Parameters			FEA Performance Responses			
	Hinge Angle, *ϴ* (°)	Strut Thickness, *t* (mm)	Strut Length, *l* (mm)	Radial Resistive Force, RRF (N)	Chronic Outward Force, COF (N)	Foreshortening, FS (%)	Peak Stress, PS (MPa)
1.	15	0.20	6.00	5.12	3.99	−0.092	445.98
2.	25	0.20	4.50	11.05	6.32	−0.142	442.00
3.	15	0.50	4.50	51.07	29.03	−0.056	418.80
4.	25	0.50	6.00	16.51	10.48	−0.166	448.93
5.	20	0.50	5.25	26.19	15.73	−0.099	429.02
6.	20	0.20	5.25	5.73	3.89	−0.126	441.01
7.	20	0.35	4.50	15.79	9.16	−0.098	427.25
8.	15	0.35	5.25	17.98	10.74	−0.065	432.21
9.	25	0.35	5.25	7.93	5.13	−0.169	445.49
10.	20	0.35	6.00	3.22	2.99	−0.119	442.59
11.	15	0.20	4.50	18.91	10.74	−0.061	430.95
12.	25	0.20	6.00	2.09	1.87	−0.256	452.12
13.	25	0.50	4.50	29.70	17.00	−0.120	433.95
14.	20	0.35	5.25	9.51	6.09	−0.110	434.77
15.	15	0.50	6.00	37.41	22.25	−0.063	433.89

**Table 4:** Actual and predicted values of mechanical performance metrics at the optimum level.

Optimal Design Parameters			Performance Responses	Actual Results by FEA	Prediction Results by RSM	Error, %
Hinge Angle (°)	Strut Thickness (mm)	Strut Length (mm)				
15	0.5	4.50	RRF (N)	51.07	50.8697	0.39
			COF (N)	29.03	28.8562	0.60
			FS (%)	−0.056	−0.0592	5.71

**Table 5:** Comparison of mechanical performance metrics for PPR, ZPR, and NPR stent structures based on [52].

Materials and Methods				Structural Designs	Radial Stiffness (MPa)	Foreshortening (%)	Publication
Material	Strut Thickness (mm)	Stent Diameter (mm)	Radial Deformation				
Elastoplastic	0.4	18	25%	ZPR	68.96	−0.84	Proposed
				PPR	32.50	7.75	[52]
				NPR	100	−51.8	

## Data Availability

Data will be available on request.

## Abbreviations

The following abbreviations are used in this manuscript:

PAD	Peripheral artery disease
ZPR	Zero Poisson’s ratio
PPR	Positive Poisson’s ratio
NPR	Negative Poisson’s ratio
FEA	Finite element analysis
RSM	Response surface methodology
ANOVA	Analysis of variance
RRF	Radial resistive force
COF	Chronic outward force
FS	Foreshortening
PS	Peak stress
